# Characteristics of Neural Network Changes in Normal Aging and Early Dementia

**DOI:** 10.3389/fnagi.2021.747359

**Published:** 2021-11-22

**Authors:** Hirohisa Watanabe, Epifanio Bagarinao, Satoshi Maesawa, Kazuhiro Hara, Kazuya Kawabata, Aya Ogura, Reiko Ohdake, Sayuri Shima, Yasuaki Mizutani, Akihiro Ueda, Mizuki Ito, Masahisa Katsuno, Gen Sobue

**Affiliations:** ^1^Department of Neurology, Fujita Health University, Toyoake, Japan; ^2^Brain and Mind Research Center, Nagoya University, Nagoya, Japan; ^3^Department of Integrated Health Sciences, Nagoya University Graduate School of Medicine, Nagoya, Japan; ^4^Department of Neurosurgery, Nagoya University Graduate School of Medicine, Nagoya, Japan; ^5^Department of Neurology, Nagoya University Graduate School of Medicine, Nagoya, Japan; ^6^Aichi Medical University, Nagakute, Japan

**Keywords:** resting state network (RSN), anatomical networks, MRI, aging, dementia, network hub, energy failure

## Abstract

To understand the mechanisms underlying preserved and impaired cognitive function in healthy aging and dementia, respectively, the spatial relationships of brain networks and mechanisms of their resilience should be understood. The hub regions of the brain, such as the multisensory integration and default mode networks, are critical for within- and between-network communication, remain well-preserved during aging, and play an essential role in compensatory processes. On the other hand, these brain hubs are the preferred sites for lesions in neurodegenerative dementias, such as Alzheimer’s disease. Disrupted primary information processing networks, such as the auditory, visual, and sensorimotor networks, may lead to overactivity of the multisensory integration networks and accumulation of pathological proteins that cause dementia. At the cellular level, the brain hub regions contain many synapses and require a large amount of energy. These regions are rich in ATP-related gene expression and had high glucose metabolism as demonstrated on positron emission tomography (PET). Importantly, the number and function of mitochondria, which are the center of ATP production, decline by about 8% every 10 years. Dementia patients often have dysfunction of the ubiquitin-proteasome and autophagy-lysosome systems, which require large amounts of ATP. If there is low energy supply but the demand is high, the risk of disease can be high. Imbalance between energy supply and demand may cause accumulation of pathological proteins and play an important role in the development of dementia. This energy imbalance may explain why brain hub regions are vulnerable to damage in different dementias. Here, we review (1) the characteristics of gray matter network, white matter network, and resting state functional network changes related to resilience in healthy aging, (2) the mode of resting state functional network disruption in neurodegenerative dementia, and (3) the cellular mechanisms associated with the disruption.

## Introduction

Patients with dementia have limitations in activities of daily living due to impaired memory, language comprehension, executive function, visuospatial function, and judgment. These functions also decline with normal aging, but without limitations in activities of daily living. This is because “intelligence,” the ability to act purposefully and with reason as well as effectively interact with the environment to solve problems, is well-preserved (Deary, 2012; Park and Bischof, 2013). Interestingly, primary sensations (hearing, touch, and smell), motor function, memory, calculation, and intuition peak at around 20 years of age, followed by a gradual decline; concentration and emotional cognition peak at around 45 years of age, while comprehension, vocabulary, and judgment peak at around 60 years (Hartshorne and Germine, 2015). Considering the aging population, relationships between memory and aging should be determined.

Alzheimer’s disease (AD), dementia with Lewy bodies (DLB), and frontotemporal lobar degeneration (FTLD) develop in the elderly population and share the accumulation of pathological proteins (tau and amyloid-β in AD, α-synuclein in DLB, and TDP-43, tau, and FUS in FTLD). However, many older adults with brain atrophy demonstrate normal higher mental functions and intelligence. The accumulation of tau and amyloid-β in AD patients begins more than 20 years before the disease onset, and there is a long asymptomatic or pre-clinical period despite accumulation of pathological proteins (Jack et al., 2013; Masters et al., 2015). In addition, some patients with mild cognitive impairment (MCI) return to the preclinical stage of the disease (with memory impairment but no limitations in activities of daily living) (Ding et al., 2016). In contrast, some asymptomatic patients have pathological accumulation of proteins that is suggestive of AD (Snowdon, 2003).

To understand the plasticity and adaptation of brain, the following facts should be considered:

1. Various aspects of intelligence peak after the age of 45 years,
2. Pathological protein accumulation and brain atrophy do not always correlate with the symptoms, and
3. Patients may transition between the preclinical and MCI stages.

Although various recent studies have provided perspectives to answer these questions in terms of the balance between energy demand and supply in the brain (Camandola and Mattson, 2017; Mattson and Arumugam, 2018; Vandoorne et al., 2018; Diederich et al., 2019), these studies mainly focused at the cellular level, and only a limited number of studies have focused on the relationship between energy and large-scale networks in the brain.

Techniques to analyze brain networks using magnetic resonance imaging (MRI) (Suárez et al., 2020), magnetoencephalography and electroencephalography (Prichep, 2007; de Haan et al., 2009) have become widely available in recent years, which has improved our understanding of the relationships between aging and dementia. Measures of structural and functional brain connectivity using MRI have become widely used as reliable indicators in assessing brain reserve and incompatibility, and in predicting progression to dementia (Farb et al., 2013; Teipel et al., 2016). In our large aging cohort study, we have also employed MRI data to investigate age-related changes in anatomical circuits (Bagarinao et al., 2018), brain volumes (Bagarinao et al., 2018), white matter fiber tracts (Choy et al., 2020), and resting-state functional circuits (Bagarinao et al., 2019, 2020a,b). We have recruited a total of more than 1,500 healthy adult volunteers aged 20–80 years from Nagoya, which is Japan’s fourth most populated city, and neighboring areas to elucidate age-related changes in brain networks. We have also collected demographic data, including education, past medical history, medication, drinking and smoking habits, and family history of neurodegenerative diseases, Mini-Mental State Examination (MMSE) and Addenbrooke’s Cognitive Examination-Revised (ACE-R) for general cognition. ACE-R evaluates five cognitive subdomains, including orientation/attention, memory, verbal fluency, language and visuospatial ability (Mioshi et al., 2006), and has been reported to have excellent sensitivities and specificities (>0.8) for the diagnosis of MCI and dementia (Livingston et al., 2017). In addition to aging studies, we have also employed similar brain network analysis to examine network alterations in patients with neurodegenerative disorders such as AD (Yokoi et al., 2018), amyotrophic lateral sclerosis (ALS) FTLD (Ogura et al., 2019; Imai et al., 2020), Parkinson’s disease with mild cognitive decline (Kawabata et al., 2018) and others.

In this review, we summarize our findings as well as existing evidence from other studies on the characteristics of brain networks of healthy older adults and patients with early dementia and discuss the backgrounds of the potential mechanism at the cellular level of the transition from healthy aging to dementia.

## Widespread Atrophic Changes and Network Alterations in the Brain’s Gray Matter With Aging

**FIGURE 1:**
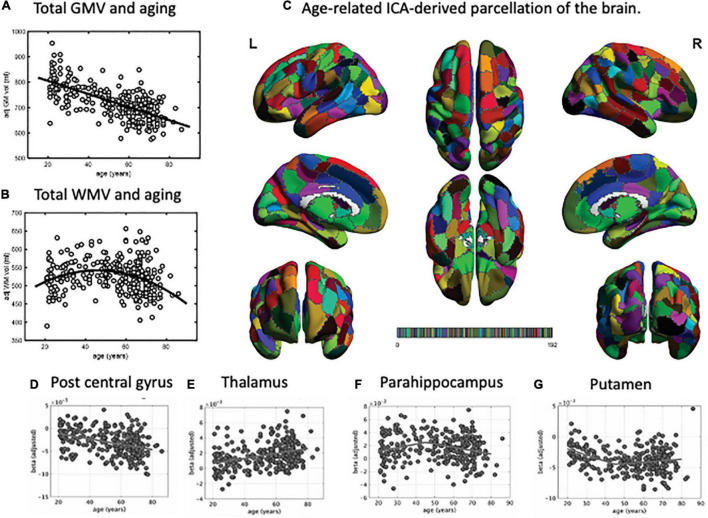
Parcellation map for the unbiased, data-driven, age-related brain structure. Total gray matter volume decreased with aging **(A)**. However, the total white matter volume peaked at around 50 years of age, followed by a decrease **(B)**. Using independent component analysis, we extracted 192 brain regions related to aging **(C)**. Based on this parcellation map, we identified regions where brain volume decreased with age (e.g., post central gyrus, **D**), remained stable (e.g., thalamus, **E**), or peaked at around 45 years of age and then decreased (e.g., parahippocampus, **F**, and putamen, **G**). GMV, gray matter volume; WMV, white matter volume; ICA, independent component analysis. This figure is adapted from Bagarinao et al. (2018).

Several MRI studies measuring brain atrophic changes with aging have shown a global decline in gray matter volume (Figure 1A; Good et al., 2001; Allen et al., 2005; Smith et al., 2007; Bagarinao et al., 2018). However, the rate of gray matter volume decline across the whole brain varies. In one study (Bagarinao et al., 2018), we identified regions with similar patterns of age-related brain atrophy in a data-driven approach using independent component analysis (ICA), a method that can be used to separate multivariate signals into multiple additive components, assuming that the observed data is a linear superposition of independent components (Figure 1C; Bagarinao et al., 2018). We have identified 192 regions, which interestingly closely resembled that of the conventional anatomical structures and Brodmann’s brain map areas. Among these regions, the volume of 174 (90.6%) negatively correlated with age, especially the central posterior gyrus (Figure 1D), cerebellum, central gyrus, and inferior frontal gyrus. On the other hand, the thalamus (Figure 1E), regions located in the medial frontal lobe, and the superior frontal gyrus demonstrated relatively preserved volumes. In addition, the volumes of regions such as the parahippocampal gyrus (Figure 1F) had an inverted U-shape relationship with age, with the maximum volume at 45–50 years. A similar U-shaped relationship was also observed with the volume of the putamen (Figure 1G).

In the same study, we also examined the relationships between age and gray matter network properties, including integration and efficiency, using graph theory. We used the ICA-identified regions as network “nodes,” the correlations of gray matter values between nodes as “edges,” and the entire brain as a set of vertices and edges that form a network. Estimated hub indices, which indicate overall brain integration, and the indices associated with network efficiency exhibited a U-shaped or inverted U-shaped relationship with age, with a peak at around 45–50 years of age. These spatially connected intrinsic brain volume changes and network reconfiguration associated with the increase in network segregation and integration after 50 years of age may provide the needed maintenance of normal social and cognitive status of the elderly and may also lead to the onset of age-related neurodegenerative diseases.

## Aging and Changes in White Matter Volume and Network

Studies investigating age-related changes in the brain’s white matter have shown that the total white matter volume had an inverted U-shape relationship with age, characterized by an initial increase in volume until around 45–50 years of age, followed by a decrease (Figure 1B; Allen et al., 2005; Fjell et al., 2013; Bagarinao et al., 2018). Aside from the total volume, more recent studies have also examined age-related microstructural changes in the white matter’s fiber tracts. Diffusion-weighted MRI (dMRI) is a technique based on the diffusion of water molecules in tissues and can be used to evaluate white matter fibers. Using dMRI, scalar quantities such as diffusion anisotropy (fractional anisotropy, FA) and mean diffusivity (MD) can be estimated and used as indirect measures of white matter integrity. Generally, FA decreases with age, while MD increases, indicating a general decline of white matter integrity with age. These metrics, however, do not consider the influence of crossing and kissing fibers in a voxel, which could affect the analysis (Jeurissen et al., 2013).

**FIGURE 2:**
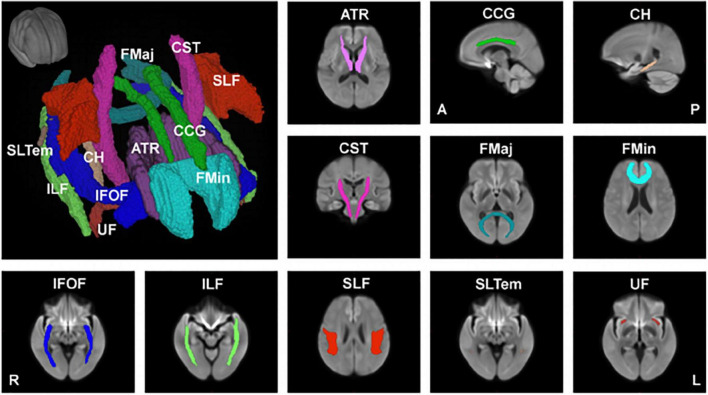
Major anatomical tracts of the brain. Major fiber tracts based on the Johns Hopkins University – International Consortium for Brain Mapping (JHU-ICBM) atlas, warped onto the study’s population template space were used for the tract-level analyses of fiber density (FD), fiber cross-section (FC), and combined measure of fiber density and cross-section (FDC). ATR, anterior thalamic radiation; CCG, cingulum (cingulate gyrus); CH, cingulum (hippocampus); CST, corticospinal tract; FMaj, forceps major; FMin, forceps minor; IFOF, inferior fronto-occipital fasciculus; ILF, inferior longitudinal fasciculus; SLF, superior longitudinal fasciculus; SLTem, superior longitudinal fasciculus (temporal); UF, uncinate fasciculus. This figure is reproduced with permission from Choy et al. (2020).

In another study (Choy et al., 2020), we used the recently developed fixel-based analysis (FBA) (Raffelt et al., 2017), which employed a more advanced diffusion model to resolve multiple fiber populations in a single voxel, to evaluate specific structural changes in each nerve fiber tract. Using FBA, we examined the relationships between age and FBA parameters, such as fiber density (FD), fiber cross-section (FC), and the combined measure of fiber density and cross section called FDC (FD × FC), in 293 healthy participants aged 21–86 years (Choy et al., 2020). We also performed tract-level analysis using the Johns Hopkins University (JHU) white matter tractography atlas, which consists of 11 major fiber tracts, including the anterior thalamic radiation (ATR), cingulum (cingulate gyrus, CCG), cingulum (hippocampus, CH), corticospinal tract (CST), forceps major (FMaj), forceps minor (FMin), inferior fronto-occipital fasciculus (IFOF), inferior longitudinal fasciculus (ILF), superior longitudinal fasciculus (SLF), superior longitudinal fasciculus (temporal, SLTem), and uncinate fasciculus (UF) (Figure 2).

Our findings showed that FD decreased significantly with age in 5 of the 11 major fiber tracts (ATR, CCG, CH, FMin, and SLF). This decrease is consistent with previous histopathological reports that white matter axons in the corpus callosum are damaged with age (Hou and Pakkenberg, 2012). On the other hand, the FD of 6 of the 11 fiber tracts did not show a significant negative correlation with age, and the FD of FMaj, IFOF, and ILF only showed a small, but insignificant, change with age. Interestingly, the FC of CH significantly increased with age, whereas that of ATR and UF showed an increasing trend with age but not significant. This may be explained by the fact that the white matter volume peaks at around 45–50 years of age, and the volumes of the middle frontal gyrus, thalamus, parahippocampal gyrus, and superior frontal gyrus remain relatively preserved. A recent meta-analysis reported that aerobic exercise could prevent the age-related decrease in hippocampal volume (Firth et al., 2018). The relationships between exercise and the associated changes in white matter fibers and brain volume during healthy aging still require further investigation. FDC, representing a comprehensive index that evaluates both FD and FC, negatively correlated with age in 6 of the 11 fiber tracts (ATR, CCG, CST, FMin, IFOF, and ILF). As for the CCG and FMin, which are mainly located in the frontal lobe, the FD, FC and FDC were negatively correlated with age.

Age-related changes in white matter fibers are not uniform, as is the case with brain volume, and about half of the major white matter circuits, especially those located posteriorly, are unaffected by aging, which may relate to the preserved cognitive function and intelligence.

As shown, some gray matter areas and white matter volumes did not uniformly decrease with age, and the volumes of white matter and some gray matter regions were not only considerably maintained until 45–50 years of age, but also tended to increase. These volume changes have important implications for the pathogenesis of various cognitive functions and intelligence in middle-aged and older adults.

In monkeys, the decrease in gray matter volume was a result of decreased dendrites and synapses, rather than neurons (Page et al., 2002), which explains why a U-shaped relationship does not occur during their aging process. A recent study has demonstrated that myelin fragmentations were gradually released from aging myelin sheaths and were subsequently cleared by microglia resulting in the formation of insoluble, lipofuscin-like lysosomal inclusions in microglia (Safaiyan et al., 2016). In a study that quantified the density of HLA-DR-positive activated microglia in the white matter of postmortem samples of cognitively normal young adults, cognitively normal older adults, and “super-agers” aged 80 years or older whose memory test scores were equivalent to or higher than those of people aged 50–65 years, the statistical significant differences in microglia density were evident between normal young adults and cognitively normal old but not between normal young adults and SuperAgers (Gefen et al., 2019). A more recent study identified white matter-associated microglia, which form in a TREM2-dependent but APOE-independent manner in aging white matter, where they form nodules that are engaged in phagocytosing damaged myelin (Safaiyan et al., 2021). These findings suggest that there are dynamic changes in the white matter of the brain that regenerate and manage degenerated myelin during aging, which may be associated with healthy cognitive function and toward disease progression.

## Age-Related Changes in the Brain’s Functional Networks

Resting-state functional MRI (rsfMRI) is a non-invasive imaging modality that can measure spontaneous low-frequency fluctuations in the blood oxygenation level-dependent (BOLD) signal. The BOLD effect is a phenomenon in which local neural activity changes the binding of blood hemoglobin to oxygen, resulting in a change in magnetism in the same region, which in turn causes a change in the MRI signal (Ogawa and Lee, 1990). Using rsfMRI, various resting-state networks (RSNs) representing spatially distinct areas of the brain that demonstrate synchronous BOLD fluctuations at rest can be identified (Biswal et al., 1995). Typical methods for studying RSNs include ICA, seed-based analysis, and graph theory. These methods examine the relationships between specific regions and the whole brain. ICA for the whole brain is useful to identify large-scale networks, such as the default mode (which activates when the participant is concentrating on an internally oriented task related to cognitive function), primary information processing-related (motor sensory, visual, and auditory networks) and multisensory integration (executive control, salience, and dorsal attention networks) networks.

In our analysis of large-scale brain networks of normal individuals (Bagarinao et al., 2019), within large-scale network connectivity was significantly reduced with age, whereas increased connectivity was observed with regions outside the network. General higher mental functions, assessed by the ACE-R score, were higher for patients with preserved connections within primary information processing-related networks such as auditory and primary visual networks.

In addition, older adults demonstrated a stronger connectivity in the dorsal attention network (which belongs to the multisensory integration network, is active during spontaneous attention and reorientation to unexpected events, and integrates multiple primary information processing), primary information processing-related network (sensorimotor and primary visual networks), default mode network (precuneus network), and the multisensory integration network (salience network). The strength of connections among the default mode, executive control, and salience networks (also called the neurocognitive networks) correlated with higher general cognitive function. On the other hand, stronger connections of the primary information processing networks (sensorimotor, primary visual, and higher visual networks) with the salience and basal ganglia networks were associated with lower general cognitive function.

An analysis of the integrity of large-scale networks in elderly and young participants (<30 years of age) demonstrated decrease in the integrity of most large-scale networks with age. Interestingly, cognitive functions were higher in participants with preserved integrity of the primary visual, higher visual, sensorimotor, and dorsal attention networks.

Using a parcellation map that subdivides the brain into 499 regions of interest (Shirer et al., 2012), we also performed network analysis using graph theory with each region of interest used as a node, and functional connections between nodes considered as edges. Our findings showed that the shortest path length decreased, the overall efficiency increased, and the network integration increased with age.

We further evaluated whether connectivity measures associated with resting-state functional networks or age affects the ACE-R scores, using mediation analysis, and observed that changes in a resting-state functional network had a stronger influence on ACE-R scores compared to age. Recent studies have demonstrated that an enhanced connectivity between resting-state functional networks plays a vital role in patients with preserved motor and higher brain functions in adulthood, despite hemisection of the brain in childhood (Kliemann et al., 2019). Additionally, in patients with glioma in the left hemisphere, enhanced executive control network on the right side was associated with preserved higher mental functions (Maesawa et al., 2015). These results support the idea that resting-state functional network connectivity is related to cognitive function.

Overall, these findings suggest that intra-network connectivity decreases and extra-network connectivity increases in human large-scale functional networks with age, and the integrity of many large-scale networks diminishes in older age. These findings are consistent with those reported previously from Europe and the United States (Betzel et al., 2014a,b), and across different ethnicities. Additionally, the connectivity of dorsal attention (Ptak et al., 2017) and large-scale networks related to primary information processing enhanced with age. However, it is interesting that the general cognitive function is better if the integrity of primary information-related network is preserved. Epidemiological studies have reported hearing loss in middle age and lack of exercise are observed in 9% and 3% of patients with dementia, respectively (Livingston et al., 2017), and reduced contrast sensitivity is a risk factor for cognitive decline in women (Ward et al., 2018). It is essential to clarify the relationships between risk factors for dementia related to primary information processing networks and resting-state functional networks. Because the strength of connections between networks plays a central role in cognitive function [such as executive control, salience, and default mode (Menon, 2011) and ACE-R scores], lifestyle habits that promote increased connectivity between these networks may help prevent dementia.

## Dementia and Functional Networks: Positioning of Hubs

As described above, healthy aging is characterized by a decline in the function of primary information processing-related networks, and retention and enhancement of multisensory integration networks. The multisensory integration network is considered the “hub” of the brain because it integrates the dispersed neural activities, thereby allowing efficient cognitive functions (Stam, 2014; Lynn and Bassett, 2019). Outside the multisensory integration network, cerebral hubs exist mainly in the default mode network (Bagarinao et al., 2020b). Hubs that integrate large networks with different functions are also called connector hubs, which are robust to aging (Bagarinao et al., 2020a), and may compensate for the loss of function of subordinate networks. In other words, connector hubs may provide robustness and resilience to the healthy aging brain.

The existing data suggest that selective hub vulnerability is responsible for the preferential accumulation of Aβ in the medial hubs of the default mode network and the preferential accumulation of tau in medial temporal lobe hubs in preclinical AD (Yu et al., 2021). In our study of early AD patients with significant amyloid-β deposition demonstrated by positron emission tomography (PET), the relationships between the distribution of THK5351 PET accumulation (reflecting tau burden), inflammation, and the resting-state functional network disruption were examined (Yokoi et al., 2018). Tau and inflammation accumulated in the precuneus (Utevsky et al., 2014) and posterior cingulate gyrus (Leech and Sharp, 2014), which are the hubs of the default mode network; the associated network disruption correlated with disease onset.

Similar findings were observed in patients with FTLD (Ogura et al., 2019; Rittman et al., 2019; Chen et al., 2020; Imai et al., 2020). We also studied patients with ALS, which is thought to reflect the early stage of FTLD. In these patients, the severity of the deficits was associated with a decrease in functional connectivity between the right spindle and lingual gyrus (the hub of speech production) as well as reduced semantic memory and word recognition (Ogura et al., 2019). In addition, we examined the decision-making disorder characteristic of FTLD, using probability inversion learning, and found that significantly more ALS patients selected a decision-making style that seemed to go their way. The degree of abnormality was associated with decreased functional connectivity of the anterior cingulate gyrus/frontal pole, a typical hub (Imai et al., 2020).

In a study of PD without dementia, which is closely related to DLB, patients with mild amnesia had impairments in the precuneus and posterior cingulate cortex, corresponding to the hub of the default mode network (Kawabata et al., 2018; Nagano-Saito et al., 2019). Intriguingly, Kanel et al. (2020) performed PET with [18F]-fluoroethoxybenzovesamicol ([18F]-FEOBV), a radioligand for vesicular acetylcholine transporter (VAChT), in patients with DLB to determine cholinergic vulnerability topography. The results showed that cholinergic vulnerability in DLB consists of important neural centers involved in the networks of tonic arousal (cingulate gyrus), attention (insular cortex), visual attention (visual thalamus), and spatial navigation (limbic and corpus callosum). This study showed that disruption of brain hub function may be associated with the development of dementia, even at the neurotransmitter level.

## Possible Cellular Mechanisms of Hub Vulnerability in the Aging Brain and Neurodegeneration

Hubs are presumed to be rich in synapses, including those on long axons. It is believed that 64% of the energy used by the brain is spent on synaptic transmission (Sengupta et al., 2013), and genes related to ATP synthesis and metabolic regulation are expressed in a coordinated manner in the mouse hub region (Fulcher and Fornito, 2016). Glucose metabolism is also active in the human hub region (Tomasi et al., 2013). On the other hand, the number and function of human mitochondria decrease by about 8% every 10 years (Short et al., 2005). Therefore, in advanced age, the hub region is always on the verge of an energy crisis. Importantly, the hub region was the preferred site of lesions of early-stage neurodegenerative dementia in our study.

Although the etiology of neurodegenerative diseases remains a mystery, there is much consensus on the effects of reduced energy metabolism, excitotoxicity, and oxidative damage on their pathogenesis (Beal, 2005; Sas et al., 2007; Fan et al., 2017; Toda et al., 2017). Regarding energy metabolism, the human brain consumes about 20% of the biological energy, even though its volume is only about 2% of the body mass (Mink et al., 1981; Attwell and Laughlin, 2001). Neurons are in a post-mitotic excitatory state and require very high energy to generate and transmit action potentials, release neurotransmitters at synapses, set static gradients of ion concentration, dispose of soluble proteins, and remove metabolites (Rangaraju et al., 2019). In addition, the metabolic cost of performing and maintaining neural functions is very high, especially for the billions of incessant synaptic transmissions, which use 80% of the energy required for the functioning of the neuronal network (Rangaraju et al., 2019). For this reason, impaired energy metabolism is considered an important trigger in aging of the central nervous system (Błaszczyk, 2020).

Oxidation of glucose, which is a fast generator of ATP, is the most important energy source for the brain, and the mitochondrial oxidative phosphorylation system plays a major role (Schönfeld and Reiser, 2013). On the other hand, the number and function of mitochondria are known to decline with age (Short et al., 2005), and mitochondrial dysfunction leads to decreased ATP production, decreased glycolysis, increased oxidative stress, limited neuronal self-repair capacity, and excessive neuronal apoptosis (Grimm and Eckert, 2017; Wang et al., 2020). Accumulation of damaged mitochondria is a universal feature of aging, and increased oxidative damage to mtDNA, mitochondrial lipids, and proteins correlates with the accumulation of dysfunctional, damaged organelles (Mecocci et al., 1993; López-Otín et al., 2013). These sequence of events can be most likely to affect neurons in the cortex, hippocampus, and basal ganglia, which have long unmyelinated axons, numerous synaptic connections, and high energy metabolism (Camandola and Mattson, 2017; Mattson and Arumugam, 2018; Vandoorne et al., 2018; Diederich et al., 2019).

In addition, accumulation of reactive oxygen species (ROS) as well as damaged mitochondria has been observed in the aging brain (López-Otín et al., 2013), and the age-related increase rate of 8′-hydroxy-2′-deoxyguanosine (OH8dG) in the cerebral cortex is more pronounced in mtDNA and a significant 15-fold increase was observed in those aged 70 years and older (Mecocci et al., 1993). ROS are involved in immune response, inflammation, synaptic plasticity, learning, and memory, and their excessive production causes oxidative stress, protein and DNA damage, and lipid peroxidation reactions (Kishida and Klann, 2007; Marinho et al., 2014). Mitochondria, in their hyperactivity, also induce excessive ROS production. Excitotoxicity is known to be associated with many pathological conditions such as stroke, epilepsy, hearing impairment due to exposure to excessive noise, and neurodegenerative diseases (Armada-Moreira et al., 2020). Under normal conditions, antioxidant enzymes such as superoxide dismutase, glutathione peroxidase, glutaredoxin, thioredoxin, and catalase regulate the levels of ROS. However, with aging, nitration and oxidation of proteins progresses, and decreased activity of SOD, catalase, and GSH reductase, as well as decreased GSH have been reported (Venkateshappa et al., 2012).

Autophagy is a fundamental cellular process that promotes homeostasis, differentiation, development, and survival by degrading molecules and intracellular elements such as nucleic acids, proteins, lipids, and organelles via lysosomes. In health and aging, autophagy plays a multifaceted role, including protein homeostasis, regulation of macromolecular availability, mitophagy, ER-phagy, nucleophagy, lysophagy, and xenophagy. Recently, the impairment of autophagy in aging and in neurodegenerative diseases has also received much attention. However, the relationship between autophagy and aging and diseases is very complex and has not yet been fully clarified (Aman et al., 2021).

Fluorodeoxyglucose (FDG) PET studies have shown that the hub region of the brain requires a lot of energy (Tomasi et al., 2013). FDG PET studies have also shown that the hub region of the brain is rich in genes related to energy (Fulcher and Fornito, 2016). While the load on the hub region increases due to age-related disturbances in the primary information processing network, energy disturbances, mainly mitochondrial disturbances, can disrupt the energy balance and oxidative stress balance in the hub region, resulting in a combination of increased ROS and disruption of autophagy. The process of dementia development, including the accumulation of pathological proteins, may be accelerated. In AD, PD, and FTLD, abnormalities in energy, mitochondria, excitotoxicity, reactive oxygen species, and autophagy beyond normal aging have been reported, and many reports indicate that these may cause accumulation of pathological proteins alone or in combination (Grimm and Eckert, 2017; Wang et al., 2020). In addition to the fact that aging is the most important risk factor for neurodegenerative diseases, it is urgent to elucidate the series of pathological conditions caused by the disruption of brain energy metabolism.

The dendritic spine of the synapse is the site of excitatory synaptic input, and is the basis for synaptic plasticity, including long-term potentiation and depression. The dendritic spine requires large quantities of ATP to support the rapid membrane pumping activity required to restore the ion gradient after synaptic activation. Therefore, in cases where the ability of a neuron to generate sufficient ATP is impaired (e.g., aging, ischemia, or neurodegenerative diseases), synapses are vulnerable to dysfunction and degeneration (Harris et al., 2012). In neurodegenerative diseases, increased activity occurs in various networks and neurons before the onset of disease, and acts as a compensatory mechanism (Gregory et al., 2017). Compensation by the hub, which is hierarchically located at the upper level of the disrupted lower network, may increase the energy requirements of the same region, and contribute to energy failure and accumulation of pathological proteins (Saxena and Caroni, 2011; de Haan et al., 2012). Treatment with febuxostat and inosine increased blood hypoxanthine and ATP in healthy adults, and a preliminary trial in 30 patients with Parkinson’s disease demonstrated symptomatic improvement (Watanabe et al., 2020).

## Limitations and Future Directions

In this review, we discussed the relationship between aging and brain network changes on individuals with normal cognition assessed mainly by general cognitive function test such as ACE-R. Since the current definition of dementia is the limitation of daily life activities due to the decline of cognitive functions, the use of general cognitive function tests to define “normal” general cognitive function will be sufficient in delineating the boundary between healthy aging and dementia. However, decline in episodic memory is a typical clinical manifestation of AD, but it is also observed early in normal aging. Interestingly, different neurocognitive processes were known to develop to compensate for this decline both in early AD and in normal aging (Tromp et al., 2015). Among different types of episodic memories, autobiographical episodic detail generation has been reported to be a good indicator for capturing minor cognitive dysfunction associated with AD (Grilli et al., 2018). Spatial navigation has also been reported to better reflect the early pathology of AD (Lithfous et al., 2013). Furthermore, processing speed, mental flexibility, digit span memory test, visual scanning, motor processes, and so on, are some of the tests that can be used to extensively evaluate the cognitive effects of aging (Hartshorne and Germine, 2015). And these indicators which are closely related to intelligence and wisdom acquired by humans with aging (Deary, 2012; Park and Bischof, 2013), may be related to the U curve phenomenon observed in gray matter and white matter volume analysis of the brain, as well as the enhancement of efficiency observed in functional networks identified using graph analysis. Changes in the default mode network have been reported even in cognitively healthy older adults, which some believe reflect processes that have enhanced cognitive function and promoted social and emotional well-being and stability in life (Andrews-Hanna et al., 2019). Changing the framework for interpreting alterations in internal cognitive function with aging may shed important light on the neurocognitive mechanisms that distinguish between normal and pathological aging and provide a more complete picture of the complexity of the aging brain. Large scale prospective observational studies with specific tasks which provide the information of early cognitive, social, and behavioral changes are essential to elucidate the relationship between healthy aging and dementia considering the inter-hemispheric and intra-hemispheric interaction and gender differences.

The inverse U-shaped change in white matter volume between the ages of 40 and 50 has been shown in several papers including ours. However, the mechanism is not fully understood. It has been shown that the peak of human cognitive function varies depending on its content. Elucidation of the relationship between these age-dependent peaks in cognitive function and gray matter, white matter, and functional circuits may provide important insights into whether the inverted U-shaped changes are compensatory or not. On the other hand, we may just be looking at the results of white matter aging and its response to changes at the molecular and cellular levels. Interestingly, Pichet Binette et al. (2020) used large multicohort structural MRI data from young to cognitively preserved elderly to derive morphological networks using ICA and extracted gray matter volumes in each derived network. In their study, the preservation of whole-brain gray matter patterns was associated with a lower risk of developing cognitive impairment than the preservation of gray matter volume.

## Conclusion

**FIGURE 3:**
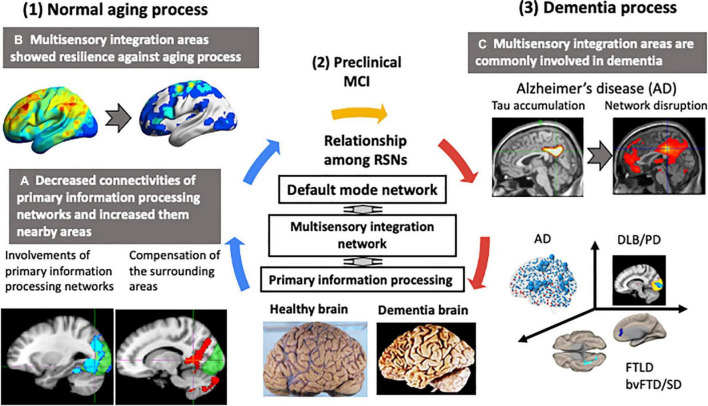
Changes in functional networks with normal aging and dementia. Neurodegenerative dementias are commonly associated with impairments in the connector hub. In the resting state networks (RSNs) of the brain, a multisensory integration network connects each primary information processing network to the default mode network. The multisensory integration networks are closely connected to each other. In normal aging, the functional connectivity of the primary information processing networks decreased and connectivity of the surrounding regions centered on the multisensory integration networks increased. The functional connectivity of the multisensory integration networks was well-preserved with age. In contrast, in dementia, hub regions, such as the multisensory integration networks and the default mode network, were the preferred sites of lesions. Early-stage Alzheimer’s disease was characterized by tau accumulation and disrupted functional connectivity in the posterior cingulate gyrus and precuneus, which are the hubs of the default mode network. This vulnerability of the hub region to disruption was also seen in Parkinson’s disease with mild cognitive decline and in amyotrophic lateral sclerosis with behavior disorder and semantic impairment.

Figure 3 summarizes the differences in functional networks between normal aging and dementia. Our basic premise is that age-related changes in brain structure and function are widespread and yet elderly participants remained cognitively normal. This suggests high degree of resilience in the brain, which possibly drives functional reorganization with hub regions playing critical roles. Structurally, there is extensive gray matter atrophy, but the rate of atrophy is not uniform across the entire brain. Similar changes can also be observed in the major white matter fiber tracts, with the fiber density exhibiting an overall decrease and fiber cross-section in some areas exhibiting an increase with age. Despite these structural changes, general cognitive performance in the elderly remains well-preserved, suggesting a high degree of resilience in the brain. This resilience may be responsible for the changes observed in the functional architecture of the brain. With aging, there is reduced connectivity within the large-scale functional networks. However, aging is also accompanied by increased connectivity of large-scale networks with outside regions, which could potentially serve as an important compensatory mechanism. Many regions, including integrative hub regions, may compensate to maintain normal cognitive function. Therefore, well-functioning hub regions may be critical for the normal general cognitive performance during healthy aging (karoshi – “death from overwork”). Failure of hub regions to compensate, due to inadequate energy supply or accumulation of pathological proteins, could trigger a cascade of network dysfunction, leading to cognitive impairment and/or dementia.

## Author Contributions

HW contributed to the acquisition and analysis of data, study supervision and coordination, review of manuscript, and study concept and design. EB contributed to the review of manuscript, interpretation of data, drafting/revising manuscript for content, study coordination and sample analyses, and authoring first draft of manuscript. SM contributed to the acquisition and analysis of data, acquisition, analysis, or interpretation of data, scientific oversight, identification and recruitment of subjects, advice on sample analysis and sample handling, review of data, and review and edit of manuscript. KH contributed to the acquisition and analysis of data, identification and recruitment of subjects, advice on sample analysis and sample handling, review of data, and review and edit of manuscript. KK and AO contributed to the acquisition and analysis of data, interpretation of data, and study coordination and sample analyses. RO contributed to the acquisition and analysis of data, and study coordination and sample analyses. SS, AU, and MI contributed to the review of manuscript and interpretation of data. YM contributed to the review of manuscript, interpretation of data, and edit of manuscript. MK contributed to the review of manuscript, review of data, and edit of manuscript. GS contributed to the study supervision and coordination, review of manuscript, study concept and design, and study supervision. All authors contributed to the article and approved the submitted version.

## Conflict of Interest

The authors declare that the research was conducted in the absence of any commercial or financial relationships that could be construed as a potential conflict of interest.

## Publisher’s Note

All claims expressed in this article are solely those of the authors and do not necessarily represent those of their affiliated organizations, or those of the publisher, the editors and the reviewers. Any product that may be evaluated in this article, or claim that may be made by its manufacturer, is not guaranteed or endorsed by the publisher.
